# Which characteristic of Natto: appearance, odor, or taste most affects preference for Natto

**DOI:** 10.1186/1880-6805-31-13

**Published:** 2012-05-28

**Authors:** Yuki Tsumura, Aki Ohyane, Kuniko Yamashita, Yoshiaki Sone

**Affiliations:** 1 Junshin Junior Collage, Tsukushigaoka, Fukuoka, Japan; 2 Department of Food and Nutrition, Osaka City University, Sumiyoshi, Osaka, Japan

## Abstract

**Background:**

In Japan, consumption of Natto, a fermented bean dish, is recommended because of its high quality protein, digestibility in the gut and its preventive effect on blood clot formation due to high vitamin K content. However, consumption of Natto in Kansai and the Chugoku area (the western part of Honshu) is less than that in the other areas of Japan probably because of a “food related cultural inhibition”. In this study, we determined which characteristic of Natto (appearance, odor or taste) most affect subjects’ perception of sensory attributes by observation of brain hemodynamics in relation to subjects’ preference for Natto.

**Findings:**

In this experiment, we defined each subject’s changes in brain hemodynamics as (+) or (−) corresponding to an increase or a decrease in total hemoglobin concentration after stimuli compared to that before stimuli. As a result, there was no relation between preference for Natto and change in brain hemodynamics by the stimuli of “looking at” or “smelling”, while there was a significant relationship between preference and stimulus of “ingestion”; (+) : (−) = 21:15 in the subjects of the “favorite” group and (+):(−) = 30:7 in the subjects of the “non-favorite” group (*P* = 0.034).

**Conclusion:**

This result indicated that characteristic “taste” of Natto most affects preference for Natto.

## Introduction

Aspects of recent human nutritional evolution have been a bio-cultural process in which learned cultural practices are critical to a wide acceptance of particular food products [1]. This bio-cultural process may cause non-uniform distribution of food preferences between traditionally and culturally different areas in a population. In Japan, this is getting smaller but there are still differences in some food preference. For example, consumption of Natto and taro in Kansai and the Chugoku area is less than that in the other areas of Japan, while consumption of bread and Worcester sauce shows a countertrend [2]. As Natto is produced by fermentation of steamed soybeans with a bacillus, it contains high quality proteins and large amounts of bacterial menaquinone 7 (MK-7, a bioactive form of vitamin K2). Because of these nutritional advantages, dieticians and Natto producers have campaigned for greater consumption of Natto in Kansai and the Chugoku area, but its consumption is still less than that in the other areas.

Questionnaires asking the reason for non-preference of Natto indicated that its appearance, odor or taste is unbearable for many people. About the factors influencing individual food choice, a number of researchers have proposed models to describe the relationship between those factors and food choice. In these models, food appearance, food odor and food texture are categorized into “intrinsic factors”, “characteristics of the food” and “perception of sensory attributes” [3], and are recognized as very important factors that influence individual food choice. In this experiment we examined which characteristic of Natto - appearance, odor or taste - most affects perception of sensory attributes by monitoring brain hemodynamics in order to find the most offensive factor for the people who answered the questionnaire unfavorably with regard to Natto consumption.

From a human physiological perspective, food preference is largely determined by the central nervous system that is stimulated by food “taste”, a concept which includes the sensations of taste, aroma, texture and appearance as well as the pleasure response to foods. In this research area, Pecina showed that sweet foods activate some specific brain pathways [4] and Levine discussed the relationship between hedonic aspects, neuroregulation and energy balance [5]. Recently, neurobiological technique has been improved and applied to a neuroregulatory study of human hedonic response; for example, near-infrared spectroscopy (NIRS) is non-invasive and widely applied in the study of the physiological index of comfort or stressful mental states (for example, Tsunetugu and Miyazaki [6]). In this connection, Ogata reported that near-infrared spectroscopy (NIRS) can be useful for the analysis of mental states during mental focus tasks and that they found an increase in the amount of total hemoglobin in the frontal cortex during stressful tasks but a decrease during healing tasks [7].

In this paper, we applied NIRS to find the most offensive factor for the people who answered the questionnaire unfavorably with regard to Natto consumption by a comparison of hemodynamic changes between the Natto favorite subjects’ group and the Natto non-favorite subjects’ group.

## Subjects and methods

We asked 156 university students (aged 19 to 24, male 80, female 76) about Natto preference with a questionnaire as candidates for the experiment in advance. Among the 156 candidates for the experiment, we grouped 40 subjects into a “non-favorite” group (20 males and 20 females), who answered that “I dislike it very much” or “I dislike it” to the question “Do you like Natto?” We grouped 40 subjects into a “favorite” group (17 males and 23 females), who answered that “I like it very much” or “I like it” to the same question. The study protocol was presented to and approved by the Ethical Committee of the Faculty of Science of Living, Osaka City University, and each participant gave informed consent for this experiment.

**Figure 1:**
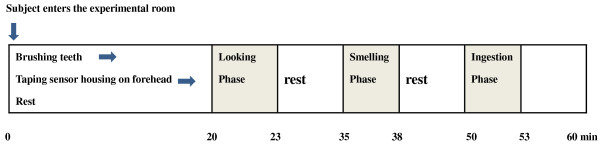
Experimental protocol.

The subjects were required to avoid eating strong smelling foods 24 hours before participation in the experiment, and to go to bed before 11 o’clock on the night of the day before the experiment day to get enough sleeping time for the morning examination. Figure 1 shows the protocol of the experiment. Every experiment was carried out in the morning, from 9:00 to 12:00. The room temperature, humidity and lighting intensity were controlled and set at 25°C, 60% and 50 lx, respectively. Two hours before coming to the experiment room every subject at the same kind of cookie and beverage as a snack, which had been given to them by an examiner the day before participation in the experiment. Just after entering the room, subjects brushed their teeth with water to make their oral cavity clean enough for smelling and ingestion of Natto before hemodynamics recording. An examiner placed the housing of optodes (the housing holds optodes of emitting and detecting of near-infrared light and the distance of the two optodes was 4 cm) on the subject’s left and right forehead, and then the subject was allowed to stay calm to become accustomed to the room atmosphere for about 10 minutes. Brain hemodynamics were monitored during three examination phases of 3 minutes with an interval of 12 minutes rest. The three examination phases were as follows: first in the “looking at phase”, second in the “smelling phase” and lastly in the “ingestion phase”. In the “looking at phase”, the participant looked at a Natto sample in a small Styrofoam container tightly wrapped in Saran Wrap for two minutes after one minute’s eye closure; in the “smelling phase”, an examiner stirred the Natto sample with chopsticks 5 cm before the subject’s nose and the subject smelled the Natto odor for two minutes (during this phase, the subjects kept their eyes closed); in the “ingestion phase” an examiner placed six grains of Natto and the subject kept them on his or her tongue for 10 seconds, rolled them in his or her mouth for 10 seconds, chewed the Natto grains for 10 seconds, then swallowed them (during this phase, the subject kept their eyes closed). Natto samples were purchased commercially and the same brand was selected throughout the experiments. We used Natto samples for the experiments at six days before the best-before date shown on the package label.

Brain hemodynamics were monitored by NIRO-200 near-infrared spectroscopy (Hamamatsu Photonics Co., Ltd., Tokyo, Japan). NIRO-200 continuously monitors and calculates changes in the oxy-([HbO_2_]), deoxy-([Hb]), and total hemoglobin ([tHb] levels. In this experiment, we sampled the levels of [HbO_2_], [Hb] and [tHb] at a rate of one Hz and used the average of 20 datasets (20 sec) as the level just before each stimulus; therefore, changes in [HbO_2_], [Hb] and [tHb] concentration are expressed in μM as a change from 0. We selected and analyzed changes in hemodynamics obtained from the left forehead, and we defined each subject’s change in brain hemodynamics as (Δ +) or (Δ −) corresponding to an increase over 1 μM or decrease under −1 μM in [tHb] for longer than 10 sec, respectively, during the 60 sec after stimuli.

Principles and details of hemodynamic monitoring by NIRS and NIRO-200 equipment were described in the manuscripts of Elwell [8] and Shibuya [9], respectively.

Chi-squared test was used to compare the number of subjects who showed an increase in [tHb] (Δ +) or a decrease in [tHb] (Δ −) after each stimulus between the “favorite” group and “non-favorite” group. A *P* <0.05 was considered to be statistically significant.

## Results

Among 40 subjects of each “favorite” and “non-favorite” group, we recorded and analyzed 36 (male: 14, female 22) and 37 (male: 17, female 20) hemodynamic data, respectively. Table 1 shows the comparison of the number of subjects who showed an increase in [tHb] (Δ +) or a decrease in [tHb] (Δ −) after each stimulus between the “favorite” group and “non-favorite” group. The Chi-square test showed that there was a significant association in the “ingestion” phase (*P* = 0.034), while there was no significant association between food preference and change in hemodynamics in the “looking at” (*P* = 0.398) and “smelling” phase (*P* = 0.808). In the “non-favorite” group, 81.1% (30 in 37) of subjects showed an increase in [tHb], while 58.3% of (21 in 36) subjects showed an increase in [tHb] in the “favorite” group during the “ingestion” phase. On the contrary, 18.9% (7 in 37) of subjects of the “non-favorite” group showed a decrease in [tHb], while 41.7% (15 in 36) of subjects of the “favorite” group showed a decrease in [tHb] during the “ingestion” phase.

**Table 1:** **Comparison of hemodynamic changes and *P*-value of Chi-squared test**

	**looking phase**		**smelling phase**		**ingestion phase**	
	**(Δ +)**	**(Δ −)**	**(Δ +)**	**(Δ −)**	**(Δ +)**	**(Δ −)**
favorite	30	6	23	13	21	15
non-favorite	27	10	25	12	30	7
	*P* = 0.398		*P* = 0.808		*P* = 0.034	

(Δ +): increase in total haemoglobin concentration after stimuli.

(Δ −): decrease in total haemoglobin concentration after stimuli.

## Discussion

Our result indicated that in the “non-favorite” group, the ratio of the number of subjects who showed an increase in [tHb] compared to that of subjects who showed a decrease in [tHb] was significantly different from the ratio in the “favorite” group during the “ingestion” phase. Concerning the relationship between hemodynamics change measured by NIRS and human “stressful” or “comfortable” stimuli, Ogata reported that the amount of total hemoglobin in the frontal cortex increased during stressful tasks [7] and Miyazaki *et al.* described that the amount of total hemoglobin decreased by “comfortable” stimuli [10]. Based on these observations in hemodymamic changes reported by Ogata and Miyazaki, we can speculate that the ingestion of Natto produced a “stressed” feeling more frequently in the subjects involved in the “non-favorite” group than in the subjects involved in the “favorite” group; on the contrary, the ingestion of Natto produced a “comfortable” feeling more frequently in the subjects involved in the “favorite” group than in the subjects involved in the “non-favorite” group. It is very interesting that there was no significant association between change in hemodynamic and food preference during the “smelling” phase in spite of the following results obtained by the questionnaire. In the questionnaire we asked the students “which characteristic of Natto do you feel uncomfortable with? Taste, odor, texture or appearance?” (multiple answers were allowed) when we recruited the participants previous to the experiment. Among the answers from 50 participants of the “non-favorite” group, they answered that the most uncomfortable characteristic was “odor”, the second was “taste”, the third was “appearance”, and the fourth was “texture” (43, 33, 22, 20, number of the answer, respectively). The result of the questionnaire indicated that the most un-comfortable characteristic of Natto was “odor” *subjectively*, but the present experiment indicated that the subjects *objectively* (physiologically) showed the most stressed feeling (possibly uncomfortable feeling) when they took Natto grains in their mouth. If the subjects of the “non-favorite” group felt the most stressed feeling due to its “odor” as answers to the questionnaire indicated, most subjects should have shown an increase in [tHb] during the “smelling” phase (but no significant association between change in hemodynamic and food preference) . These facts suggested that the “odor” sensed by food smelling was different from the “odor” sensed by food ingestion as represented by the two English words “aroma” and “flavor”, respectively, that have a distinctly different meaning (the former means “a strong smell”, the later “the particular taste of a food”). In addition, Ganong described that in human visceral senses it is difficult to distinguish the taste of food from the smell of food because they are associated closely physiologically and the flavors of various foods are in large part a combination of their taste and smell [11]. In this connection, present “physiological” results indicated that we should consider one more factor of the taste, the characteristic slimy texture of Natto, as a trigger that caused the stressed feeling in the subjects. In order to know which factor of the taste of Natto, its flavor or slippery texture, is the most affecting trigger that caused the stressed feeling in the subjects, we should have asked the subjects “What factor of Natto made you feel uncomfortable?” just after their participation in the examination by NIRS. That there was no interview on such a question to the subjects is one of the limitations of this paper. In conclusion, the present result showed that most subjects of the “non-favorite” group felt an “uncomfortable” reaction to the “taste” of Natto (which may include its flavor and slimy texture) not to the “odor” or appearance of Natto.

## Competing interests

The authors have no conflict of interest to declare.

## Authors’ contributions

YS was a general coordinator and did the study design. YS, AO, and KY were involved in data collection, data interpretation, and result analysis and literature search. All authors contributed in preparing and are responsible for final editing and approval of the manuscript.

## References

[B1] Leonard WR Stinson S, Bogin B, Huss-Ashmore R, O’Rourke D Human nutritional evolution Human Biology, an Evolutionary and Biocultural Perspective 2000 New York: John Wiley & Sons, Inc 295 343

[B2] Honkawa Y A Pictorial Record of Social Current Condition 2011 http://www2.ttcn.ne.jp/honkawa/7756.html

[B3] Shepherd R Mela D Sandler MJ, Strain JJ, Caballero B Factors influencing food choice Encyclopedia of Human Nutrition. Volume 2 1999 London, UK: Academic 843 850

[B4] Pecina S Berridge KC Hedonic hot spot in nucleus accumbens shell: where do mu-opioids cause increased hedonic impact of sweetness? J Neurosci 2005 25 11777 11786 10.1523/JNEUROSCI.2329-05.2005 16354936 PMC6726018

[B5] Levine AS Kotz CM Gosnell BA Hedonic aspects, neuroregulation, and energy balance Am J Clin Nutr 2003 78 834S 842S 14522747 10.1093/ajcn/78.4.834S

[B6] Tsunetsugu Y Miyazaki Y Measurement of absolute concentrations of prefrontal region by near-infrared time-resolved spectroscopy: example of experiments and prospects J Physiol Anthropol Appl Hum Sci 2005 24 469 472 10.2114/jpa.24.469 16079600

[B7] Ogata H Ishi Y Murkai T Ohnishi H Yagi T Study on physiological indexes during mental focus task – comparison between near-infrared spectroscopy electroencephalography, heart rate variability and peripheral arterial tonometry –IEEJ Trans EIS 2009 129 1808 1814 In Japanese with English Abstract 10.1541/ieejeiss.129.1808

[B8] Elwell CE Cope M Edwards AD Wyatt JS Delpy DT Reynolds EO Quantification of adult cerebral hemodynamics by near-infrared spectroscopy J Appl Physiol 1994 77 2753 2760 7896617 10.1152/jappl.1994.77.6.2753

[B9] Shibuya K Ueda C Sato K Shimizu-Okuyama S Saito M Kagata A Kamo M Osada T Sadamoto T Perceived exertion is not necessarily associated with altered brain activity during exercise J Physiolo Anthropol 2009 28 63 69 10.2114/jpa2.28.63 19346666

[B10] Miyazaki Y Morikawa T Yamamoto N Effect of wooden odoriferous substances on humans Jpn J Physiol Anthropol 1999 4 special issue 49 50 In Japanese

[B11] Ganong Smell & Taste, Review of Medical Physiology 1999 19 Stamford, CT, USA: Prentice-Hall International, Inc 177 183

